# The trophoblast survival capacity in preeclampsia

**DOI:** 10.1371/journal.pone.0186909

**Published:** 2017-11-06

**Authors:** Martina Hutabarat, Noroyono Wibowo, Berthold Huppertz

**Affiliations:** 1 Postgraduate Department, Doctorate Program of Medical Science, Faculty of Medicine Universitas Indonesia, Jakarta, DKI Jakarta, Indonesia; 2 Department of Obstetric and Gynecology, Division of Maternal Fetal Medicine, Faculty of Medicine Universitas Indonesia, Jakarta, DKI Jakarta, Indonesia; 3 Institute of Cell Biology, Histology and Embryology, Medical University of Graz, Graz, Austria; Otto von Guericke Universitat Magdeburg, GERMANY

## Abstract

**Background:**

Preeclampsia has become the world’s major maternal health problem putting a huge burden on mothers, newborns and also on the health systems. The pathogenesis of preeclampsia seems to include events in very early pregnancy affecting differentiation of placental villous trophoblast. The arising changes of the cell death spectrum from apoptosis via increased autophagy and aponecrosis to necrosis in turn induce systemic inflammation of the mother.

**Methods:**

Placental tissue samples and maternal serum samples from 40 pregnant women were collected from normal pregnancy, IUGR, early-onset and late-onset preeclampsia. Immunohistochemistry for LC3B and Beclin-1 was quantified using systematic random sampling techniques. Serum levels of LDH and other markers were assessed in serum.

**Results:**

Expression of the autophagy markers LC3B and Beclin-1 was significantly different between groups as was the LC3B/Beclin-1 ratio. Early-onset preeclampsia and IUGR had the highest autophagy protein expression levels, while normal pregnancy and late-onset preeclampsia had the highest LC3B/Beclin-1 ratio. Early-onset preeclampsia had the highest negative correlation with free LDH as cell defect marker.

**Conclusions:**

Autophagy plays a critical role in the cell death spectrum and cellular survival capacity of villous trophoblast. Alterations in autophagic protein expression are involved in pathological pregnancies such as preeclampsia.

## Introduction

Preeclampsia is one of the global maternal health problems with a tremendous impact on maternal and perinatal morbidity and mortality. [1,2] The placenta is still believed to be the origin of preeclampsia. [3] Today, it is known that defects in placental development during the early stages of pregnancy are the main predisposing factors for preeclampsia leading to altered release of factors from the villous trophoblast already during the first trimester of pregnancy. [3–5] The severity of this condition is determined by maternal susceptibility and hence response to placental factors released into maternal blood. [4,6] Dysregulation of cell biological processes within the villous trophoblast of the placenta will result in complex processes that end up in a cell death spectrum characterized by apoptosis, aponecrosis, as well as autophagy as modifying factor. [5,7,8] The differences along this spectrum result in the pathological features and maternal clinical consequences. [9]

Normal turnover of villous trophoblast includes cell death regulatory actions closely resembling those of apoptosis of a single cell. [10] Processes of early apoptosis already start on the single cell level in the villous cytotrophoblasts, while late stages of apoptosis can only be found in specific areas of the syncytiotrophoblast. These stages lead to the release of apoptotic structures called syncytial knots. The release of this material steadily increases during normal pregnancy and does not harm the mother. [6]

At the same time, autophagy as fundamental biological process has a role in cellular survival mechanisms of the trophoblast as well. [11,12] Autophagy has a dynamic capacity to determine the type of cell death by modifying the respective regulatory proteins. The failure of a cell to modify autophagy processes and thus to maintain its integrity will result in a cell death spectrum between apoptosis and necrosis.[8,12,13] Disturbance of the balance of these two roles of autophagy culminates in the process of autophagy failure. The respective dysregulated processes are characterized by the increase of autophagy markers such as LC3 and Beclin-1 which play significant roles in autophagy and cell death. [14] The ratio of LC3/Beclin-1 has been proposed as a new parameter which does not only show autophagy failure but also the cellular survival capacity if looked at from the opposite perspective. [11–13] The cellular survival capacity can be regarded as the biological buffer to cope with environmental cell stress. A higher ratio value indicates better cell survival, while a lower ratio value indicates failure of cell survival. Failure of cell survival and subsequent cell death by necrosis or aponecrosis in the trophoblast layer of the placenta will induce an inflammatory response of the mother and is characterized by the release of a variety of molecules such as lactate dehydrogenase (LDH) enzymes and other factors and subcellular particles into the maternal circulation. This LDH release is commonly used as indicator of necrosis in preeclampsia. [15,16]

In this study we aimed at further defining the role of autophagy as cellular survival mechanism in the villous trophoblast in early-onset and late-onset preeclampsia in comparison with healthy term pregnancies and IUGR pregnancies.

## Materials and methods

This study included pregnant women who went to Cipto Mangunkusumo Hospital and Budi Kemuliaan Hospital, Jakarta, Indonesia, between August and October 2015. All samples were collected consecutively and divided into four groups: normal term gestation, late-onset preeclampsia, early-onset preeclampsia, and intrauterine growth restriction (IUGR) based on the following inclusion and exclusion criteria. The inclusion criteria were all pregnant women whose pregnancy was terminated by cesarean section due to early-onset preeclampsia, late-onset preeclampsia, IUGR and normal term pregnancy without complication in the labor process. Exclusion criteria were all pregnant women who suffered from heart disease in pregnancy, diabetes mellitus, autoimmune disease during pregnancy, pregnant women with congenital abnormality of the baby, preterm labor, severe infection during pregnancy and not willing to take part in this study. The sample size was determined by a mean difference formula in each group, which was 10 samples for one group. Therefore, the total number of subjects in this study is 40 with 10 subjects in the normal gestation group, 11 subjects in late-onset preeclampsia, 9 subjects in early-onset preeclampsia, and 10 subjects in the IUGR group.

The subjects were pregnant women whose delivery dates were based on obstetric medical indications. Maternal blood serum samples were taken for peripheral blood test and LDH marker at the time of patient admission in the delivery or emergency room. Samples were processed immediately afterward. All samples were processed by standardized preanalytical procedures. After delivery, placental tissues were taken and processed according to a standardized pathology protocol.

Normal gestation was pregnancy until term without any complications. Late-onset preeclampsia was defined as blood pressure increase ≥140/90 mmHg at a gestational age >34 weeks with proteinuria +1 on dipstic test. Early-onset preeclampsia was defined as blood pressure increase ≥140/90 mmHg at a gestational age <34 weeks with proteinuria +2 on dipstic test or >5 g/24h, accompanied by organ involvement such as eclampsia, lung edema, HELLP syndrome, placental abruption, acute renal failure, or intrauterine growth restriction. Intrauterine growth restriction (IUGR) was defined as fetal weight estimation lower than the 10^th^ percentile on the modified Lubchenco curve for Asian people and presence of an oligohydramnios (AFI <5 cm) image on ultrasonography.

LDH measurements were done using Advia 1800 equipment (Siemens Healthcare GmbH, Erlangen, Germany) by an automated enzymatic colorimetric method with quality controlled variation coefficient <5%. Normal values for adults are in the range of 208–378 IU/l.

Evaluation of autophagy markers LC3B and Beclin-1 was done by immunohistochemistry. A modified immunohistochemistry method was performed in this study based on a semi-quantitative measurement using systematic random sampling of images and sites of counting. This enabled us to evaluate staining intensity as well as the morphology of trophoblast. This method provided information on expression of autophagy proteins plus site of expression. This is not possible with PCR or Western blotting using tissue extracts.

Five (5) μm thick formalin-fixed paraffin-embedded tissue sections were mounted on Superfrost Plus slides (Menzel/Thermo Fisher Scientific). Sections were deparaffinized in Histolab Clear^®^ (Sanova) and rehydrated through a mixture of 100% ethanol/Histolab Clear^®^ 1:1, followed by a series of graded alcohol and finally Aqua dest, according to standard procedures.

Antigen retrieval was performed for 7 min at 120°C in a decloaking chamber (Biocare Medical) in 10 mM sodium citrate buffer pH 6.0 for anti-Beclin-1 antibody (abcam) or Epitope Retrieval Solution pH 8.0 (Novocostra, Leica) for LC3B/MAP1LC3B antibody (Novus biologicals), respectively. After cooling for 20 min at room temperature, slides were transferred into TBS including 0.05% Tween 20 (TBS/T, Merck). Sections were stained either manually or using a staining robot (Autostainer 360, Thermo Fisher Scientific). To quench endogenous peroxidase, slides were incubated with hydrogen peroxide block (Lab Vision/Thermo scientific) for 10 minutes, and after washing with TBS/T, non-specific background was blocked by incubation with Protein Block (Lab Vision/Thermo scientific) for 7 min. Primary antibodies were diluted in Antibody Diluent (Dako) as follows: Beclin-1 1:280, LC3B 1:3000. Sections were incubated for 45 min at room temperature. After washing with TBS/T, slides were incubated for 20 min with the UltraVision HRP-labelled polymer and following another washing step, the polymer complex was visualized by incubating the slides with AEC Chromogen Single Solution (Lab Vision/Thermo scientific) for 10 minutes. Following washing steps with Aqua dest, sections were counterstained with hemalaun and mounted with Kaiser’s glycerine gelatine (Merck) or Aquatex (Merck).

In order to strengthen the objectivity, three criterias of quantification methods were performed. First, serial and repeated measurements. Second, systematic random sampling and third is blindness of the person who did the counting by pseudomyzing the images. All placental examinations were performed using three (3) blocks per placenta from central and intermediate positions of each placenta. Beforehand, to assure trophoblast specific staining, Cytokeratin 7 (CK7) staining was performed on serial sections of placenta. For each block, 10 slides were made and images were taken using a Leica DM 6000B microscope with an Olympus DP 72 camera and VIS software (Visiopharm A/S) by systematic uniform random sampling. For each slide, 10 images were taken and used for quantification. These methods of serial and repeated counting were performed to strengthen the quantification objectivity. Using a point grid with 16 times 12 points (total of 192 dots) placed on each image using the newCAST software (Visiopharm A/A, Demark), each point was assigned to different categories. The categories were intervillous space, villous stroma, villous trophoblast and fibrin. Within the category villous trophoblast staining intensity was ranked 3 for the strongest staining, 2 for moderate staining, and 1 for the weakest staining or negative if the dot was placed on trophoblast without staining.

The ratio between strong and weak/no staining (also called positivity) was calculated by counting the dots with ranks 2 and 3 and dividing this count by the sum of the dots with ranks 0 and 1 within villous trophoblast. The stained trophoblast was calculated as follows: dots in villous trophoblast were ranked 0 to 3 and for each protein and image the ranking was added and divided by the number of dots in villous trophoblast. Finally, the ratio of LC3 to Beclin-1 was calculated using the two above approaches to calculate staining intensity parameters. For each parameter the two values of LC3B and Beclin-1 were divided resulting in the ratio of LC3B/Beclin-1. This ratio represents one solution for the limitation of autophagy interpretation, as it showed the cellular survival mechanism as one of the aspects of autophagy.

All data collected was statistically analyzed using SPSS v22 software. Univariat analysis was done to evaluate the subjects‘ descriptive characteristics. Bivariat One-way ANOVA or Kruskal-Wallis analysis was done to answer the difference between autophagy markers between each group while Spearman correlation test was done in order to see the corelation between variables.

Before this study was started, signed informed consent was given to the research subject and her family and ethical clearance was given from the Institutional Review Board, Health Research Ethic Commitee—Faculty of Medicine University of Indonesia and Cipto Mangunkusumo Hospital (*Komite Etik Fakultas Kedokteran Universitas Indonesia* No:668/UN2.F1/ETIK/2015) as approval.

## Results

### Characteristics

The mean age of subjects was 30.4 years with a standard deviation (SD) of 5.9 years. There were 5 women (12.5%) aged <24 years, 23 women (57.5%) aged 25–34 years, and 12 women (30.0%) aged 35–42 years.

There were 47.5% women who were primipara and 52.5% were multipara. The median was parity 2, range 1–5. Gestational age during labor had a median of 38 weeks, range 25–40 weeks. Mean infant weight was 2,418.5 g (SD 920.3 g). Infant gender distribution was 24 males (60%) and 16 females (40%). Physical examination of the women showed a mean weight of 57.4 kg (SD 12.6 kg), a mean height of 156.9 cm (SD 5.5 cm), and a mean body mass index (BMI) before pregnancy of 23.4 kg/m^2^ (SD 4.7 kg/m^2^). Subjects’ characteristics in the four groups are shown in Table 1.

**Table 1:** Subjects’ characteristics.

Characteristics	Group				Total
	Normal	Late-Onset PE	Early-Onset PE	IUGR	
	(n = 10)	(n = 11)	(n = 9)	(n = 10)	(n = 40)
Age [years] (SD)	32.3 (5.5)	29.4 (6.6)	32.6 (5.3)	27.8 (5.4)	30.4 (5.9)
Gestational age [weeks] (range)	39 (38; 40)	38 (36; 40)	32 (25; 35)	37 (31; 38)	38 (25; 40)
Parity, frequency [n]					
Primi	5	4	4	6	19
Multi	5	7	5	4	21
Weight before pregnancy [kg] (SD)	62.8 (7.1)	56.3 (15.4)	60.7 (14.9)	50.2 (9.6)	57.4 (12.6)
Height [cm] (SD)	160.6 (5.6)	155.1 (4.7)	156.3 (5.3)	155.3 (4.9)	156.9 (5.5)
BMI before pregnancy [kg/m^2^] (SD)	24.3 (2.8)	23.7 (5.4)	24.9 (6.3)	20.7 (2.9)	23.4 (4.7)
Blood pressure [mmHg] (range)					
Systolic	110 (100–140)	150 (140–190)	150 (140–240)	110 (100–140)	140 (100–240)
Diastolic	70 (60–80)	100 (90–140)	100 (90–160)	70 (70–90)	90 (60–160)
Baby gender [n]*					
Male	7	4	5	8	24
Female	3	7	4	2	16
Birth weight [g] (SD)	3,267.0 (354.5)	2,972.7 (586.7)	1,337.2 (640.6)	1,933.5 (437.2)	2,418.5 (920.3)
Growth Percentile	10–97	3–95	3–70	3–10	

Numeric data is shown as mean (standard deviation) or median (min; max); BMI = Body Mass Index; PE = preeclampsia;

IUGR = Intrauterine Growth Restriction; SD = Standard Deviation. When data showed normal distribution,

mean and SD are shown. When data were not normally distributed, median and range are shown.

*All subjects were delivered by cesarean section.

### Circulating factors in maternal blood

Significant differences of the median of lactate dehydrogenase (LDH) enzyme levels were found in the four groups. Post-hoc analysis showed that there was a significant difference between normal gestation and early-onset preeclampsia (p = 0.001). There was no significant difference for hemoglobin, hematocrit and albumin levels as shown in Table 2.

**Table 2:** Maternal laboratory test results.

Variable	Group				P value
	Normal	Late-Onset PE	Early-Onset PE	IUGR	
	(n = 10)	(n = 11)	(n = 9)	(n = 10)	
Hemoglobin [g/dl] (SD)	12.0	12.3	12.7	11.7	0.399^#^
	(1.1)	(1.5)	(1.2)	(1.3)	
Hematocrit [%] (SD)	36.0	35.9	36.7	34.4	0.688^#^
	(2.5)	(4.0)	(4.0)	(4.1)	
**LDH [IU/l] (range)**	**304** ^a^	**390**	**449** ^a^	**315**	**<0.001**^$^
	**(233; 393)**	**(274; 1,102)**	**(379; 2,608)**	**(221; 435)**	
Albumin [g/dl] (SD)	3.6	3.3	3.6	3.8	0.164^#^
	(0.4)	(0.4)	(0.3)	(0.2)	

Numeric data is shown as average (standard deviation) or median (min; max);

^#^One-way ANOVA test;

^$^Kruskal-Wallis test;

^a^: Post hoc test between normal gestation and early-onset PE (p = 0.001).

When data showed normal distribution, mean and SD are shown. When data were not normally distributed, median and range are shown.

### LC3B and Beclin-1 in placental trophoblast between groups

This study evaluated two autophagy protein markers, light chain 3B/LC3B and Beclin-1 by using immunohistochemistry in combination with serial systematic random sampling. The level of expression of both proteins was evaluated in villous trophoblast and was used as a parameter for the relation of strong versus weak/no staining (+++, ++ versus +, 0) or the positivity level (0, +, ++, +++).

There was a significant difference in the median of LC3B staining in villous trophoblast between the four groups, both for the relation of strong versus weak staining (positivity level) as well as for staining intensity (expression level). There were stronger expression levels in trophoblast of early-onset preeclampsia and IUGR compared to the median population. Post-hoc analysis for LC3B expression in villous trophoblast indicated significant differences between the normal pregnant and IUGR group (p = 0.002). LC3B positivity levels in the normal pregnant, IUGR, and early-onset preeclampsia groups were higher than the median population. Post-hoc analysis for LC3B positivity level showed a significant difference between the normal pregnant group and the late-onset preeclampsia group (p = 0.026).

There was a significant difference in the mean of Beclin-1 staining in villous trophoblast between the four groups. In the early-onset preeclampsia and IUGR groups, mean Beclin-1 staining was higer than the median population. Post-hoc analysis showed that there was a significant difference between the normal pregnant and the early-onset preeclampsia group (p<0.001) and between the normal pregnant and the IUGR group (p = 0.013).

**Fig 1:**
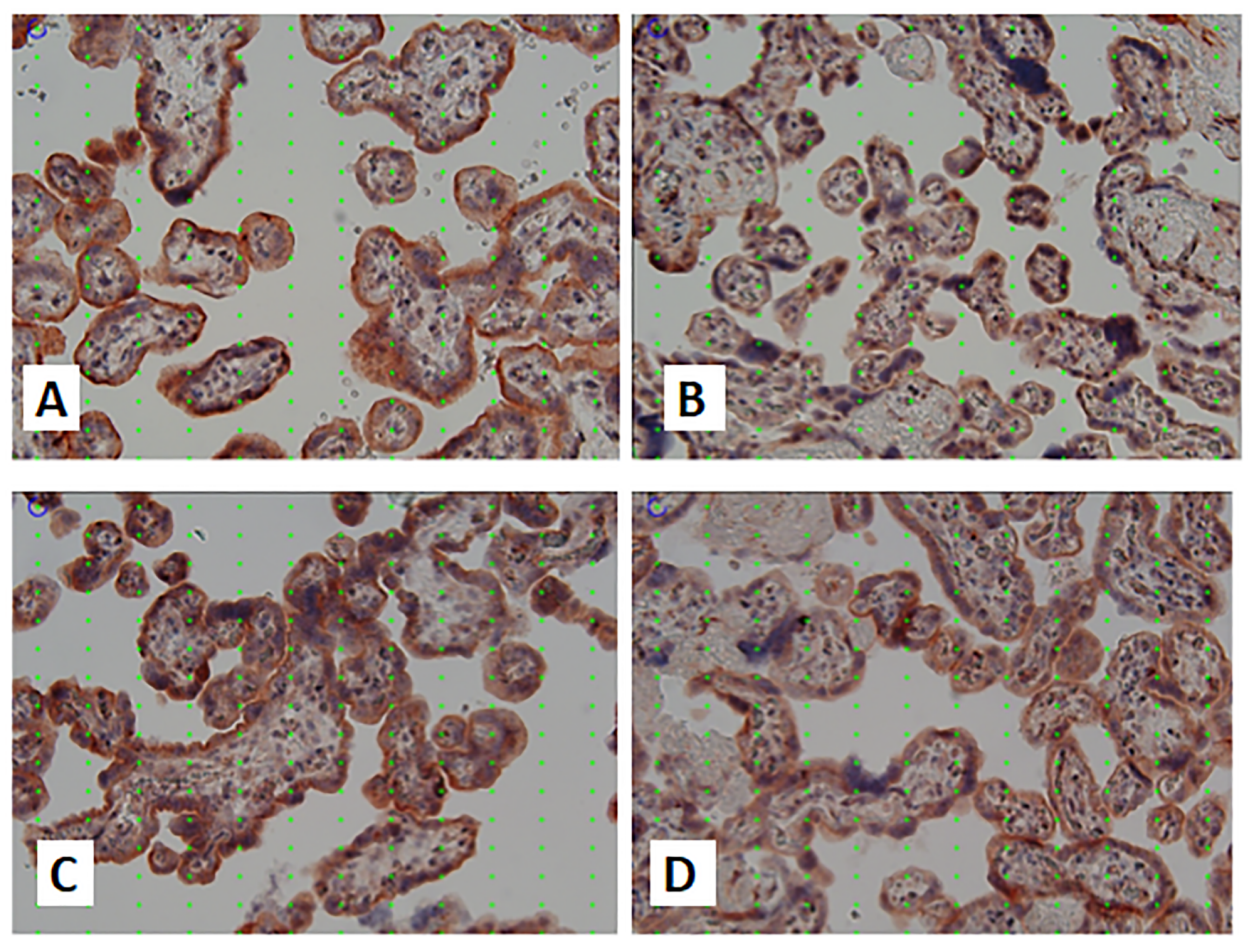
Images of LC3B staining in research groups. Placental immunohistochemical staining for LC3B in normal term pregnancy (A), late onset preeclampsia (B), early onset preeclampsia (C) and IUGR (D). Quantification was performed based on systematic random sampling. The point grid with 16 times 12 points placed on each image was used for systematic random quantification of staining There was stronger staining for LC3 in the syncytiotrophoblast in normal term pregnancy compared to late onset preeclampsia, while there was stronger staining in the syncytiotrophoblast in early onset preeclampsia compared to IUGR. The strongest staining intensity was found in early onset preeclampsia. Original magnification x200.

**Fig 2:**
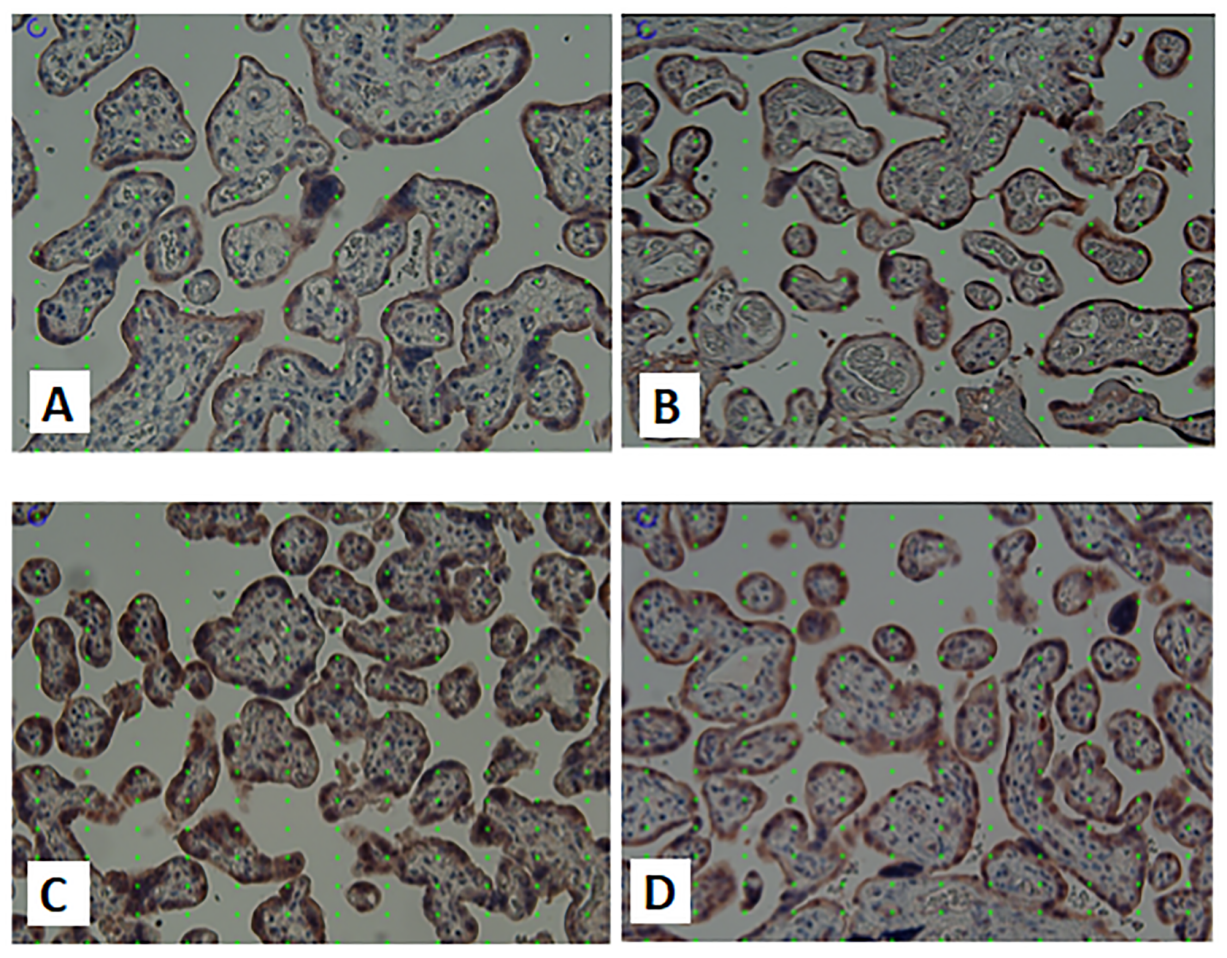
Images of Beclin-1 staining in research groups. Placental immunohistochemical staining for Beclin-1 in normal term pregnancy (A), late onset preeclampsia (B), early onset preeclampsia (C) and IUGR (D). Quantification was performed based on systematic random sampling. The point grid with 16 times 12 points placed on each image was used for systematic random quantification of staining There was stronger staining for Beclin-1 in late onset preeclampsia compared to normal term pregnancy, while there was no difference in staining intensity for Beclin-1 comparing early onset preeclampsia and IUGR. The strongest staining intensity was found in early onset preeclampsia. Original magnification x200.

There was no difference of the positivity level of Beclin-1 expression in the four groups. Early-onset preeclampsia and IUGR had median levels of Beclin-1 expression higher than the median population. The examination results based on the images of the four groups (Figs 1 and 2) are presented in Table 3.

**Table 3:** Autophagy markers in villous trophoblast.

Characteristics	Groups					p-value
	Normal	Late PE	Early PE	IUGR	Total	
	(n = 10)	(n = 11)	(n = 9)	(n = 10)	(n = 40)	
**LC3B**						
*Stained trophoblast (range)*	0.88 ^a^ (0.76; 0.94)	0.78 (0.60; 0.93)	0.92 (0.86; 0.96)	0.97 ^a^ (0.90; 0.99)	0.91 (0.60; 0.99)	< 0.001^$^
*Relation strong vs weak/no staining (range)*	1.20 ^b^ (0.12; 4.05)	0.35 ^b^ (0.08; 1.29)	0.97 (0.39; 4.30)	2.16 (0.50; 7.56)	0.97 (0.08; 7.56)	0.001^$^
**Beclin-1**						
*Stained trophoblast (SD)*	0.56 ^c^^,^ ^d^ (0.12)	0.55 (0.10)	0.84 ^c^ (0.12)	0.73 ^d^ (0.17)	0.66 (0.17)	< 0.001^#^
*Relation strong vs weak/no staining (range)*	1.04 (0.06; 5.86)	1.18 (0.29; 3.18)	3.58 (0.02; 14.61)	3.80 (0.03; 20.36)	1.80 (0.02; 20.36)	0.226^$^

Data is presented as mean (standard deviation) or median (min; max);

^#^One-way ANOVA Test;

^$^Kruskal-Wallis Test;

^a^: Post hoc test between normal pregnancy and IUGR (p = 0.002);

^b^: Post hoc test between normal pregnancy and late PE (p = 0.026);

^c^: Post hoc test between normal pregnancy and early PE (p<0.001);

^d^: Post hoc test between normal pregnancy and IUGR (p = 0.013).

When data showed normal distribution, mean and SD are shown. When data were not normally distributed, median and range are shown.

#### LC3B and Beclin-1 in placental trophoblast related to gestational age

The expression of LC3B and Beclin-1 in the normal pregnant group was lower than the total population assuming that the normal pregnancy group (gestational age 38–40 weeks) had normal autophagy processes and levels. Further analysis was done to evaluate the expression of the autophagy proteins in the three pathological groups based on gestational age respective to neonatal survival rate are shown in Table 4 below.

**Table 4:** Autophagy markers in villous trophoblast related to gestational age.

Variables	Gestational Age (week)				
	26–30	31–35	36–40	Total	p-value
	(n = 4)	(n = 9)	(n = 17)	(n = 30)	
**LC3B**					
*Stained trophoblast (SD or range)*	0.93 (0.02)	0.95^a^ (0.01)	0.88^a^ (0.60; 0.98)	0.92 (0.60; 0.99)	0.024^$^
*Relation strong vs weak/no staining (SD or range)*	1.86 (0.75)	2.91^b^ (0.77)	0.54^b^ (0.08; 2.23)	0.89 (0.08; 7.56)	0.010^$^
**Beclin-1**					
*Stained trophoblast (SD or range)*	0.92^c^ (0.68; 0.95)	0.80^d^ (0.43)	0.60^c^^,^^d^ (0.04)	0.69 (0.03)	0.001^#^
*Relation strong vs weak/no staining (SD or range)*	4.73 (1.72)	8.26 (2.54)	1.72 (0.03; 7.84)	2.33 (0.02; 20.36)	0.122^$^

Data is presented as mean (standard deviation) or median (min; max); Group 1 = 26–30 wk, group 2 = 31–35 wk, group 3 = 36–40 wk;

^#^One-way ANOVA Test;

^$^Kruskal-Wallis Test.

^a^: Post hoc test between group 2 and 3 (p = 0.01);

^b^: Post hoc test between group 2 and 3 (p = 0.004);

^c^: Post Hoc test between group 1 and 3 (p = 0.006);

^d^: Post hoc test between group 2 and 3 (p = 0,04).

When data showed normal distribution, mean and SD are shown. When data were not normally distributed, median and range are shown.

There were significant differences in median levels of LC3B protein expression. Villous trophoblast from placentas with a gestational age below 35 weeks had higher values for LC3B than the median population. For the expression of Beclin-1, there was a significant difference in staining of trophoblast, but there was no significant difference in trophoblast positivity. Beclin-1 stained trophoblast in placentas with a gestational age below 35 weeks also had higher values compared to the average population.

### The ratio of LC3B/Beclin-1

**Fig 3:**
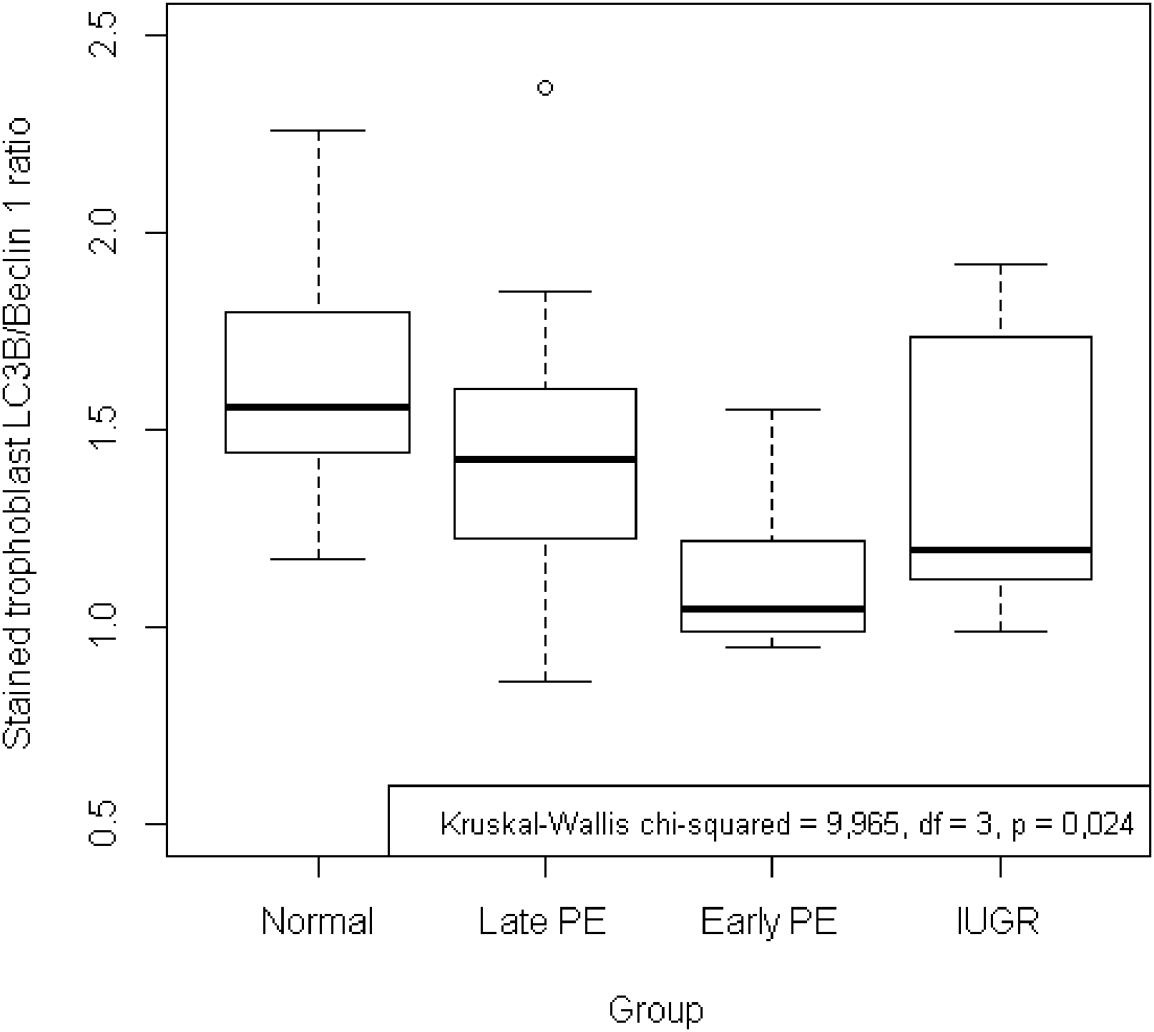
Ratio of LC3/Beclin-1 of stained trophoblast in villous trophoblast. There was a significant difference in the stained trophoblast ratio of LC3B/Beclin-1 with higher values of the ratio in normal pregnancy (p = 0.024). Post hoc analysis identified that the ratio of the normal pregnant group was significantly higher compared to the early-onset preeclampsia group (p = 0.008).

The ratio of LC3B/Beclin-1 showed the failure of autophagy or cellular survival capacity. Among the four groups, there was a significant difference in the ratio of LC3B/Beclin-1 in the parameter of stained trophoblast as shown in Table 5 and Fig 3. For the ratio of LC3B/Beclin-1 in the parameter of level of positivity, no significant differences were found between the four groups as shown in Table 5. The values of the ratio of LC3B/Beclin-1 in both parameters in normal pregnancy were higher than in the other three groups. In the post hoc test, there was a significant difference between the normal pregnant group and the early-onset preeclampsia group (p = 0.008).

**Table 5:** Ratio of LC3B/Beclin-1 in villous trophoblast.

Variables	Group					p-value
	Normal	Late PE	Early PE	IUGR	Total	
	(n = 10)	(n = 11)	(n = 9)	(n = 10)	(n = 40)	
Ratio of LC3B/Beclin-1 [Stained Trophoblast] (SD)	1.63 ^a^ (0.34)	1.46 (0.41)	1.13 ^a^ (0.20)	1.39 (0.34)	1.41 (0.37)	0.024^#^
Ratio of LC3B/Beclin-1 [Positivity] (range)	1.91 (0.04; 11.05)	0.36 (0.04; 1.63)	0.70 (0.05; 19.33)	0.62 (0.06; 57.89)	0.63 (0.04; 57.89)	0.169^$^

Data is presented as mean (standard deviation) or median (min; max);

^#^One-way ANOVA Test;

^$^Kruskal-Wallis Test;

^a^: post hoc test between normal pregnancy and early-onset PE (p = 0.008).

When data showed normal distribution, mean and SD are shown. When data were not normally distributed, median and range are shown.

**Fig 4:**
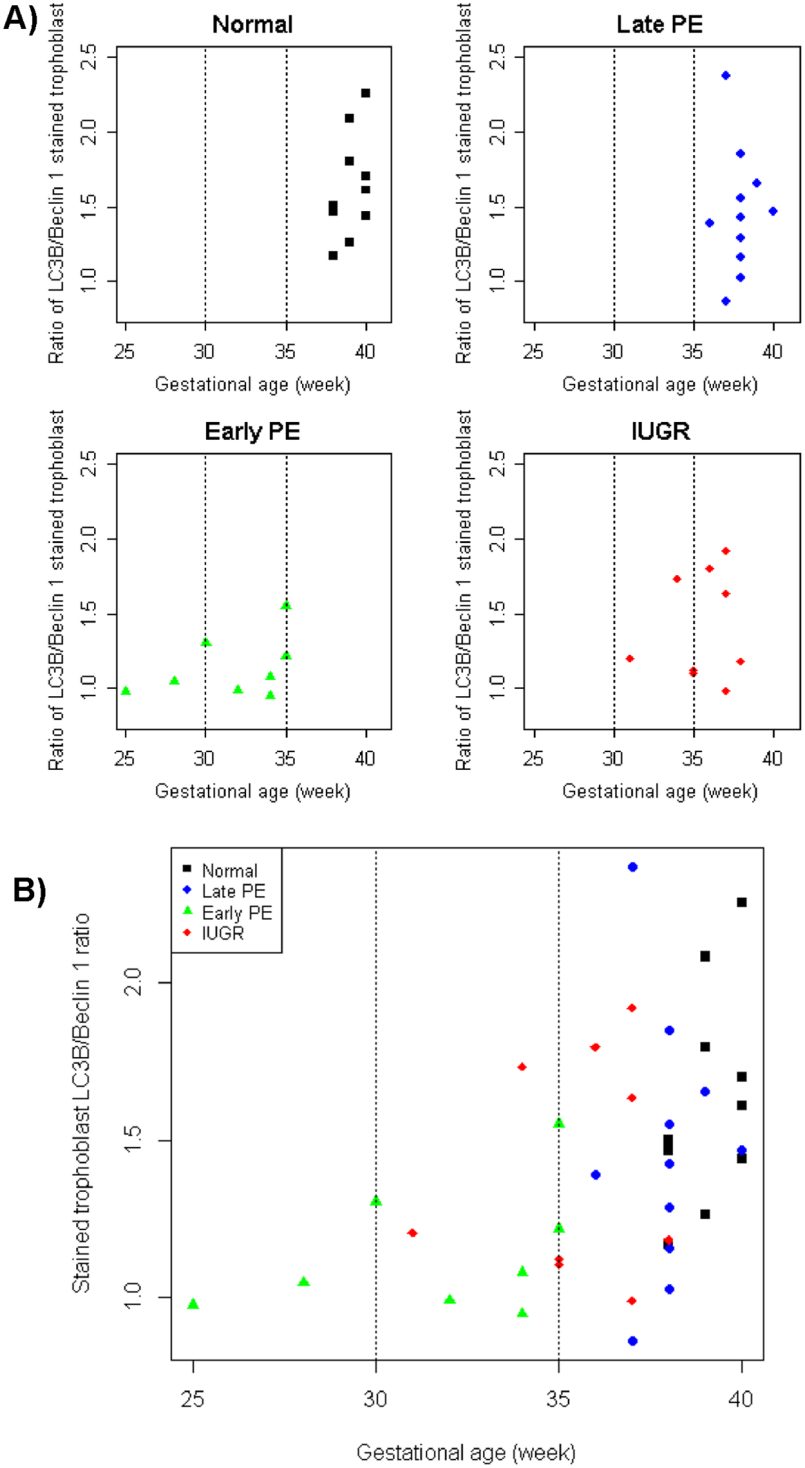
Scatter diagram showing the correlation of autophagy markers with gestational age. (a) The ratio of LC3B/Beclin-1 of stained trophoblast was correlated with gestational age for the four groups separately and (b) comparing all groups in one diagram. There was a significant difference between the four groups (p = 0.024). Analysis based on gestational age showed no significant differences of the ratio between the four groups. Separating values <35 weeks GA and >35 weeks GA revealed lower values in the early-onset preeclampsia and IUGR groups compared to the late-onset preeclampsia and normal term groups.

After exploration of the ratio LC3B/Beclin-1 in pathological pregnancies, analysis based on gestational age was performed. The results show no significant differences in both trophoblast parameters between the three gestational age groups as shown in Table 6. Compared to the total population value, villous trophoblast from placentas with a gestational age under 35 weeks had lower or equal values. Further evaluation showed that villous trophoblast in early-onset preeclampsia and IUGR had ratio values of LC3B/Beclin-1 lower than the other two groups when the gestational age was below 35 weeks as shown in Fig 4.

**Table 6:** Ratio of LC3B/Beclin-1 in villous trophoblast related to gestational age.

Variable	Gestational Age (week)				
	26–30	31–35	36–40	Total	p-value
	(n = 4)	(n = 9)	(n = 17)	(n = 30)	
Ratio of LC3B/Beclin-1 [Stained Trophoblast] (SD)	1.09 (0.72)	1.21 (0.09)	1.45 (0.09)	1.21 (0.86–2.36)	0.096^$^
Ratio of LC3B/Beclin-1 [Positivity] (range)	0.56 (0.21)	0.39 (0.05; 19.33)	0.45 (0.04; 57.89)	0.42 (0.04; 57.89)	0.865^$^

Data is shown as mean (standard deviation) or median (min; max);

^$^Kruskal-Wallis Test.

When data showed normal distribution, mean and SD are shown. When data were not normally distributed, median and range are shown.

**Fig 5:**
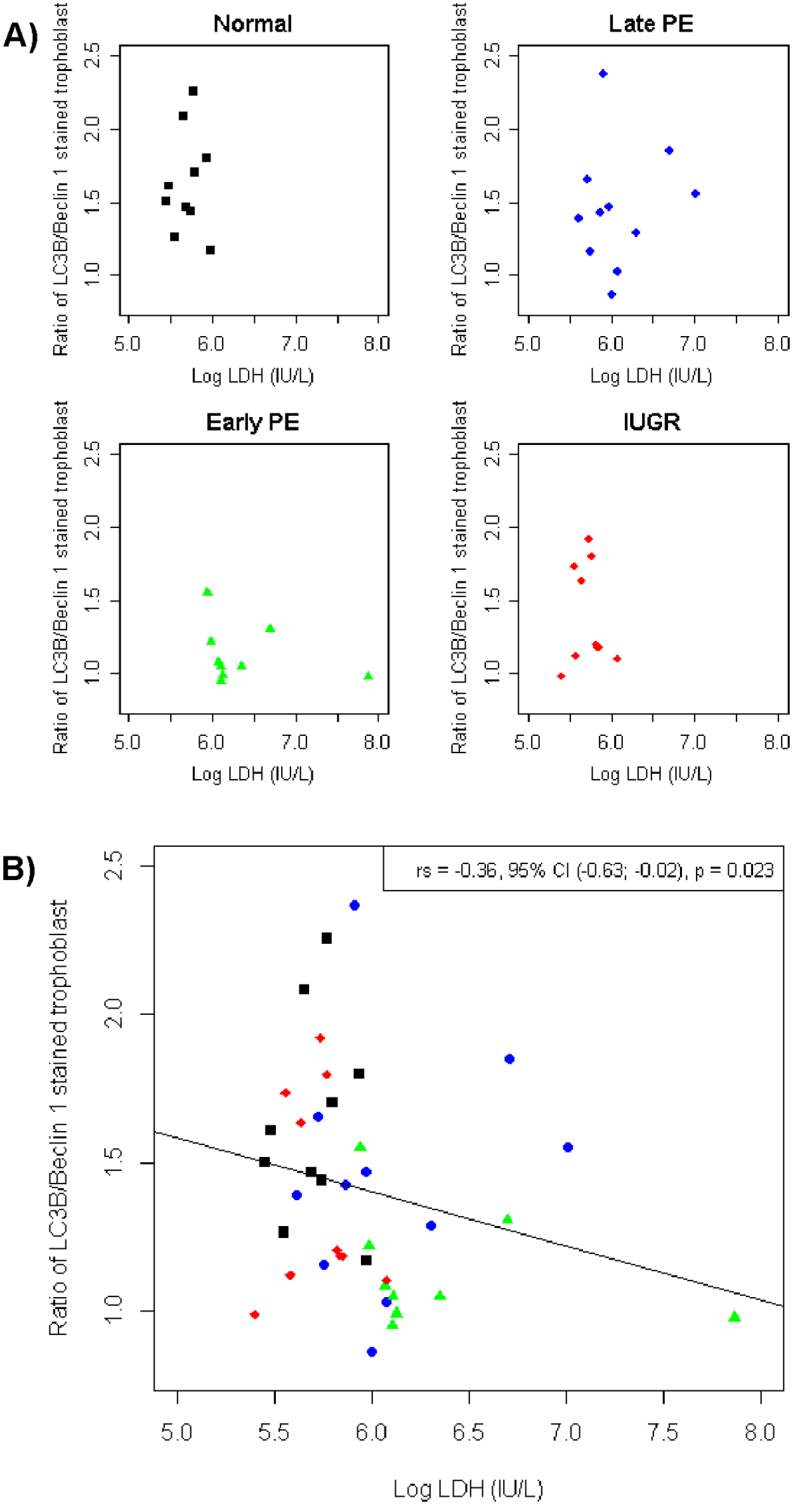
Scatter diagram showing the correlation of autophagy markers with LDH. (a) The ratio of LC3B/Beclin-1 of stained trophoblast was correlated with LDH in maternal serum for the four groups separately and (b) comparing all groups in one diagram. There was a significant difference between the four groups (p = 0.023). The early-onset preeclampsia and IUGR groups showed a negative correlation, while the normal pregnant and late-onset preeclampsia groups showed a positive correlation.

A correlation test between ratio LC3B/Beclin-1 in villous trophoblast and LDH in maternal serum showed that there was a significant difference between the four groups as shown in Table 7 and Fig 5. All of the groups showed a significant difference of correlation between the ratio of LC3B/Beclin-1 and LDH. The early-onset preeclampsia and IUGR groups showed a negative correlation, while the normal pregnant and late-onset preeclampsia groups showed a positive correlation. The early-onset preeclampsia group had the highest negative correlation among all groups.

**Table 7:** Correlation of autophagy markers with LDH.

Variables	Groups				
	Normal	Late PE	Early PE	IUGR	Total
	(n = 10)	(n = 11)	(n = 9)	(n = 10)	(n = 40)
**Coefficient of Correlation (r)**					
**Ratio of LC3B/Beclin-1** (*Stained Trophoblast*)	0.03 (p = 0.934)	0.04 (p = 0.915)	-0.45 (p = 0.224)	-0.08 (p = 0.829)	-0.36 (p = 0.023)
**Ratio of LC3B/Beclin-1** (*Positivity*)	-0.02 (p = 0.960)	-0.36 (p = 0.285)	-0.57 (p = 0.110)	-0.62 (p = 0.054)	-0.37 (p = 0.021)

Data is presented as coefficient of correlation, Spearman correlation test; LDH = Lactate dehydrogenase

**Fig 6:**
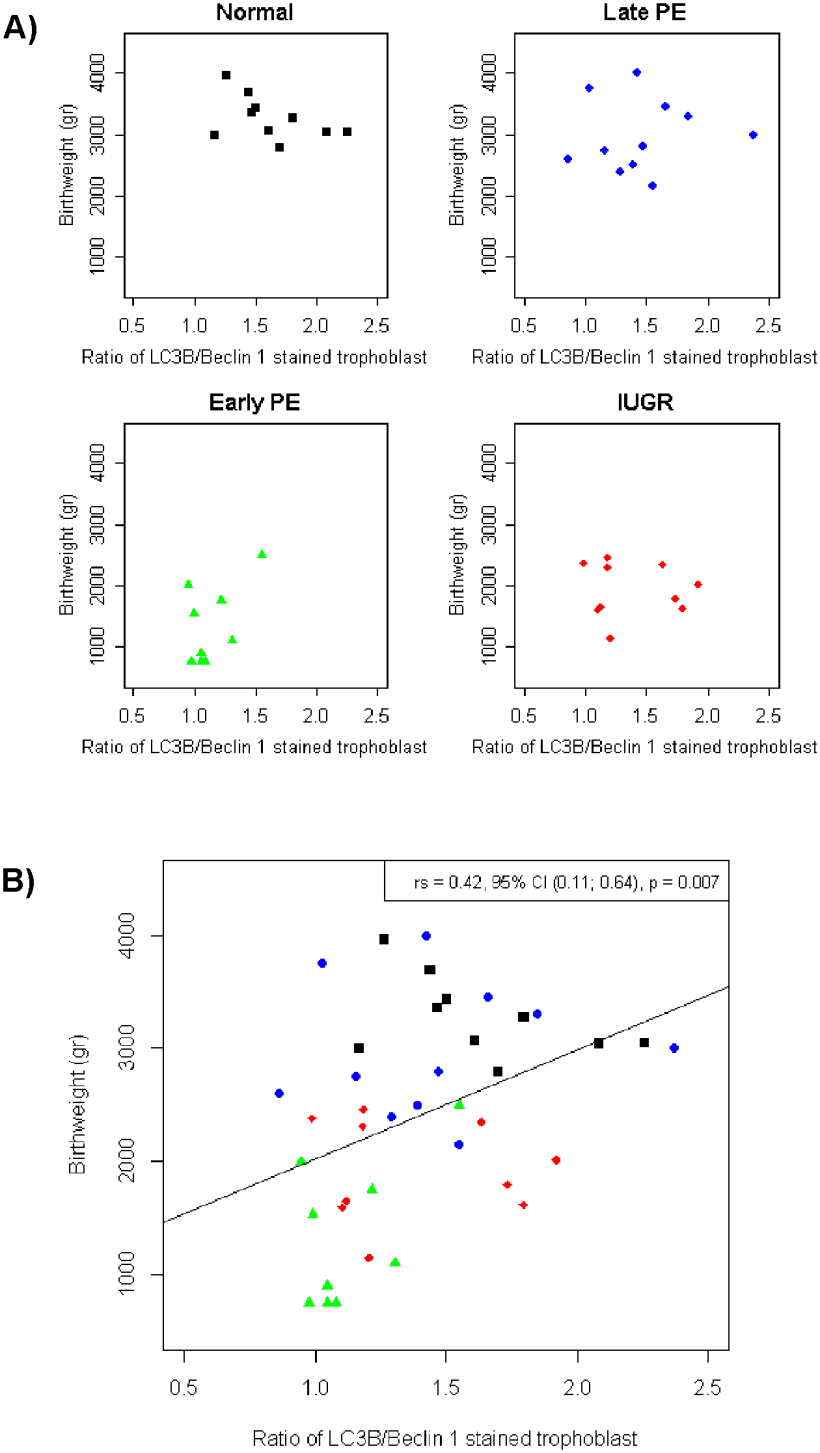
Scatter diagram showing the correlation of autophagy markers with newborns’ birthweight. (a) The ratio of LC3B/Beclin-1 of stained trophoblast was correlated with the newborns’ birthweight for the four groups separately and (b) comparing all groups in one diagram. There was a significant difference between the four groups (p = 0.007). The early-onset preeclampsia and IUGR groups showed a positive correlation, while the normal pregnant and late-onset preeclampsia groups showed a negative correlation.

A correlation test between autophagy markers in villous trophoblast and baby’s birthweight showed that there was a significant difference between the four groups (shown in Fig 6). All of the groups showed a significant difference of correlation between the ratio of LC3B/Beclin-1 and baby’s birthweight. The early-onset preeclampsia and IUGR groups showed a lower ratio with lower birthweight, while the normal pregnant and late-onset preeclampsia groups showed a higher ratio with higher birthweight. This result showed the role of autophagy as survival mechanism with nutrition as its major regulator in placental metabolism in correlation with baby’s birthweight.

**Fig 7:**
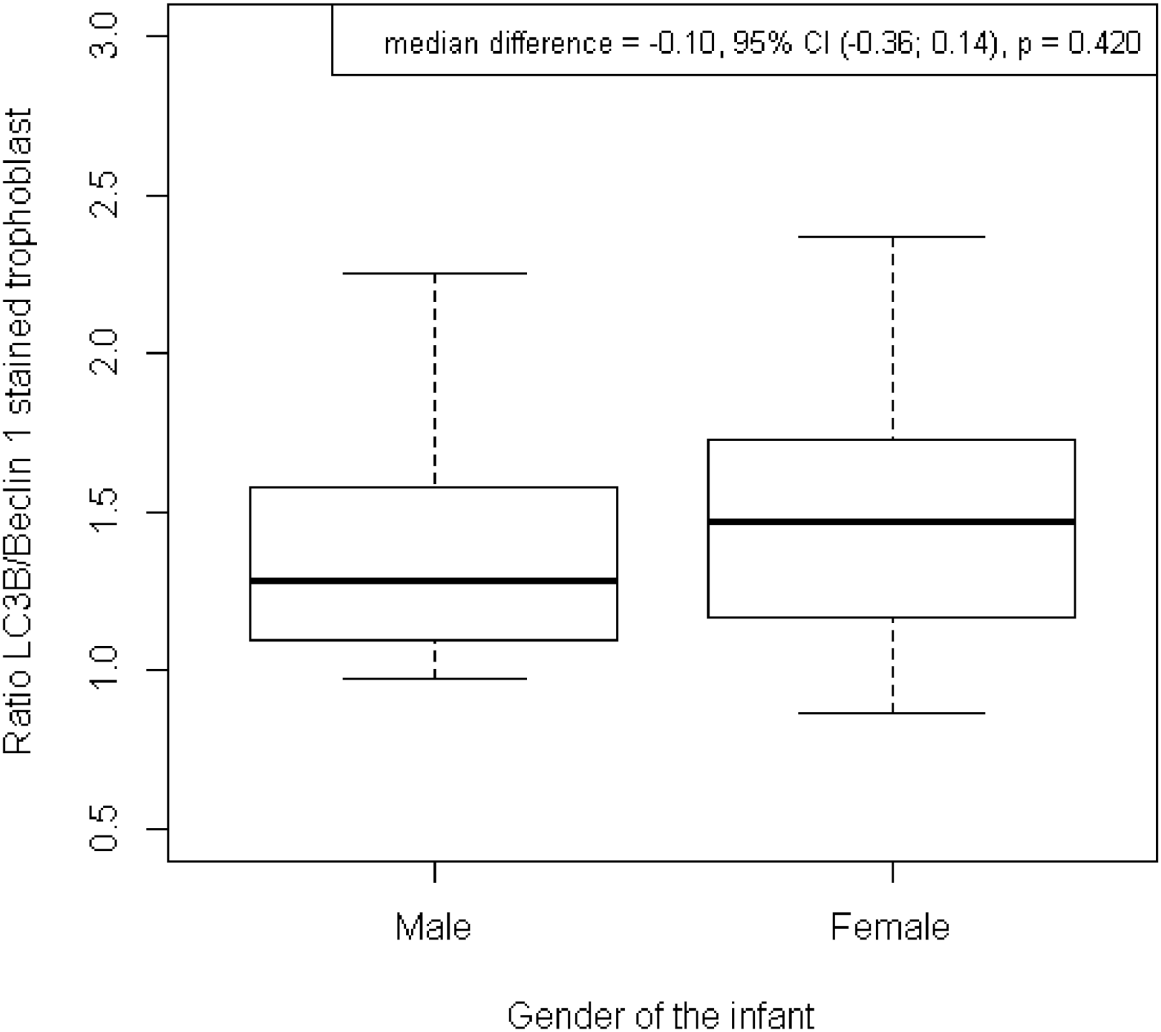
Distribution of the ratio LC3/Beclin-1 of stained trophoblast related to gender. There was no significant difference in the stained trophoblast ratio of LC3B/Beclin-1 comparing male and female newborns.

There were no significant difference in distribution of the LC3/Beclin-1 ratio related to infant’s gender. It showed that gender of the baby did not contribute to survival mechanisms of trophoblast. Both gender have the same survival charateristic (shown in Fig 7).

**Fig 8:**
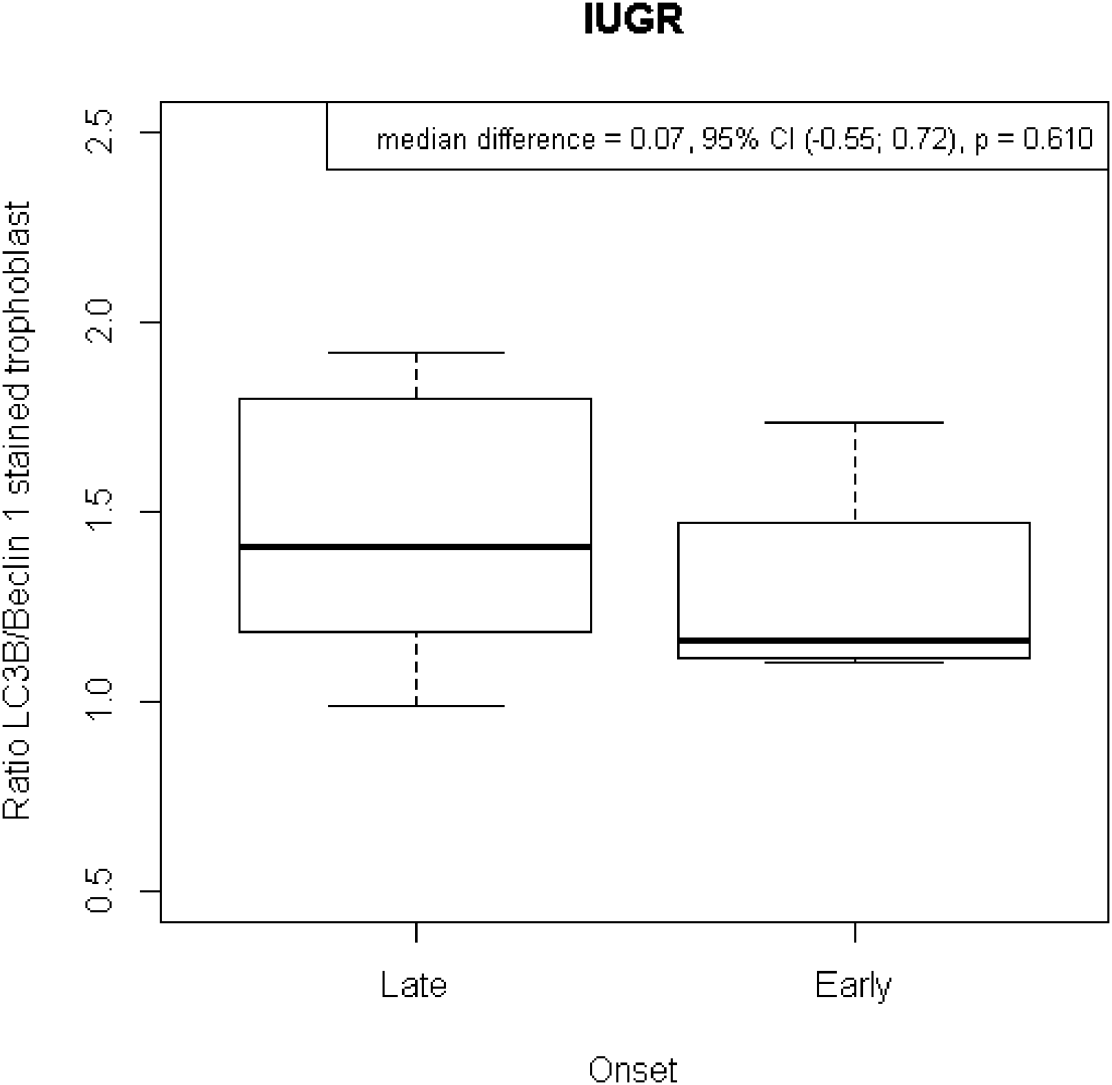
Distribution of the LC3/Beclin-1 ratio of stained trophoblast in early onset preeclampsia with or without IUGR. There was no significant difference in the stained trophoblast ratio of LC3B/Beclin-1 comparing cases of early-onset preeclampsia with and without IUGR.

There were no significant differences in the distribution of the LC3B/Beclin-1 ratio in the early onset preeclampsia group regarding presence or absence of IUGR (shown in Fig 8). It showed that in early onset preeclampsia the defect of trophoblast differentiation much more affects the maternal side rather than the fetal side. However, in late onset preeclampsia the data showed that there were 3 subjects (27,3%) had baby’s birthweight below 10th percentile. These results might showed the biological nuance of placentation defect.

## Discussion

Autophagy is a fundamental and complex biological process of living organism to maintain cell integrity.[12,13] The process begin with environment signal to Beclin-1 as part of autophagy chain process inducing either pro-death or pro-survival cascade by involvement of anti-apoptotic protein.[11] The subsequent processes are formation of autophagosome followed by fuse to lysosome formed autophagolysosome and ended up in organelles degradation to yield new energy sources.[12,13] Light chain-3/LC3 is autophagy protein which was used as marker that showed autophagy activity. While Beclin-1 involved in cross talk communication between apoptosis and autophagy.[11] Use of these two protein showed the activity of autophagy though the interpretation remains elusive.[14]

Autophagy is part of the homeodynamic system of cell death and survival and can be measured by two different methods: the static steady state and the dynamic autophagic flux.[16] There were several limitations encountered in this study. First, the assessments were only performed in a static manner at a particular time using immunohistochemistry of the placental autophagy proteins LC3B and Beclin-1. Second, placental tissue was collected at the end of pregnancy which did not allow interpretation of the dynamic and real time processes during pregnancy, as it is not possible to collect the placenta during pregnancy. Finally, the interpretation of placental autophagy activity should be assessed in a bidirectional way to capture the two aspects of autophagy, either cell death or cellular survival mechanisms to identify the dynamic processes of pregnancy.

The results showed significant differences in the expression levels of the autophagy markers LC3B and Beclin-1 revealed as stained villous trophoblast between the four groups. There were higher autophagy protein levels in early-onset preeclampsia and IUGR compared to normal pregnancy and late-onset preeclampsia. Both, LC3B and Beclin-1 autophagy markers displayed similar results in the four groups. This data let us infer that autophagy as part of the cell death spectrum was more pronounced in early-onset preeclampsia and IUGR. This result is very interesting since it bridges alterations of villous trophoblast development early in pregnancy to altered cell death pathways at the end of pregnancy. It is also concordant with previous studies showing an increase of LC3B in preeclampsia and IUGR.[17,18]

As has been hypothesized before, placentation defects at the very beginning of pregnancy may affect villous as well as extravillous trophoblast development, resulting in the development of early-onset preeclampsia and IUGR.[3] Huppertz [3] stated that inadequate trophoblast differentiation at the blastocyst stage will trigger a cascade of defects in the subsequent differentiation of villous and extravillous trophoblast, as occurring in early-onset preeclampsia and IUGR.[19] As is known, trophoblast differentiation is a tightly controlled process involving interactions between oxygen partial pressure, growth factors, transcription factors, and other signaling molecules.[17,20] Inadequate differentiation processes will trigger a series of reactions leading to failure of villous maturation, spiral artery transformation, inflow of maternal blood into the intervillous space [21,22] and also to inadequate release of particles from the villous trophoblast (necrotic and aponecrotic rather than apoptotic) resulting in immunological and inflammatory processes of the mother. This is the spectrum of cell death occurring in early-onset preeclampsia and IUGR.[3]

Looking into the processes of altered trophoblast turnover, cell demise and their relationship to autophagy as part of the programmed cell death spectrum, it is easily explainable that expression of autophagy-related proteins in early-onset preeclampsia and IUGR is higher than in the control group. This is especially true for Beclin-1. As a result of inadequate early differentiation of trophoblast during initial placentation, the immature trophoblast will be more vulnerable to environmental stress. This process may induce a chain of reactions yielding products of increased apoptosis as found in early-onset preeclampsia and IUGR. [5]

The importance to figure out the distribution of the autophagy protein across gestation was to show the autophagy activity during pregnancy. Pregnancy by physiology showed the spectrum of cell death by the formation of syncytial knots which was normal in pregnancy. As known, pregnancy itself is a dynamic process and the sufficient nutrient and energy is crucial to maintain the process. The neonatal survival ability was determined by lung maturity which was at the average age of 35 weeks. Related to gestational age, the median of autophagy protein expression was higher after 35 weeks compared to cases with delivery prior to 35 weeks. Accelerated autophagy protein expression is probably caused by the acceleration of trophoblast’s aponecrosis or premature aging. This finding goes in line with other studies showing increased apoptosis in pathological pregnancies.[23, 24]

The proteins LC3B and Beclin-1 are widely used as autophagy marker. The failure of autophagy process showed by ratio of autophagosome protein which was LC3B and Beclin-1 which was part of apoptotic cascade, based on the timing of autophagosome formation. Beclin-1 was firstly induced by the environment signal, followed by subsequent process marked by LC3B. This ratio proposed not only to show the failure of autophagy but also the cellular survival mechanism. There were significant differences in the mean values of the LC3B/Beclin-1 ratios among the four groups. The highest value was found in normal pregnancy and this was significantly different to the lower values in early-onset preeclampsia. This result shows that the worst autophagy failure occurred in early-onset preeclampsia, which was independent on the presence of IUGR in those cases. Trophoblast differentiation and maturation were thus strongly correlated with alterations in the cell death spectrum in the pathological pregnancy groups, especially in early-onset preeclampsia.[3] This has recently been highlighted by in vitro studies showing increased levels of apoptosis, aponecrosis and autophagy in cases with early-onset preeclampsia. [25]

It is tempting to speculate that the results show that there is a more severe failure of autophagy in early-onset preeclampsia and less so in IUGR and that there is stronger and/or better cellular resistance against this failure in normal pregnancy and late-onset preeclampsia. Moreover, it appears that the weaker cellular resistance in early-onset preeclampsia and IUGR associates with a failure in differentiation and maturation processes of trophoblast throughout pregnancy. This reinforces what has been explained earlier: Adequate trophoblast differentiation processes in early stages of placentation will lead to better cell survival as in normal pregnancy and late-onset preeclampsia. Furthermore, it appears that the process of trophoblast differentiation and maturation is closely related to the spectrum of cell death, as occurs in pathological pregnancies such as early-onset preeclampsia and IUGR.^3^ The above results also illustrate the role of autophagy as cellular resistance mechanism that plays a role in maintaining cellular homeodynamics.[13, 26, 27]

The same with distribution of autophagy protein across gestation; there was a tendency to increase of ratio LC3/Beclin-1 related to gestational age. The further the pregnancy age the higher the ratio was found. Although not statistically significant, it assumed the maturity of trophoblast in later gestational age.

Interestingly, this study identified the highest LDH levels in maternal blood in both preeclampsia groups. Late- and early-onset preeclampsia showed higher cell damage than normal pregnancy and IUGR. This is in line with data showing the highest release of necrotic material from cases with preeclampsia compared to normotensive IUGR and controls.[6] In this study, the ratio of LC3B/Beclin-1 was correlated with a marker of cell damage, free LDH. The correlation from data of stained trophoblast showed a significant negative correlation of 0.36 taking all subjects into account. Looking at the specific groups, normal pregnancy, IUGR and late-onset preeclampsia did not show any correlation, while only early-onset preeclampsia showed a strong negative correlation with LDH. This correlation indicates the presence of better cell survival strategies in normal pregnancy, IUGR and late-onset preeclampsia. The correlation for early-onset preeclampsia shows that the lower the ratio of LC3B/Beclin-1 (i.e. the worse cell survival is preserved) the higher the free LDH levels.[15, 28, 29]

The involvement of nutrition as major regulator in autophagy showed by the correlation of ratio LC3/Beclin-1 with baby’s birthweight. Baby’s birthweight is a resultante of the complex dynamic process of pregnancy. From the perspective of autophagy and nutrition, the result showed the significant positive corelation which was the better the survival the higher the birthweight. This was also showed the role of nutrition in autophagy process.

The above results demonstrate the involvement of autophagy in fundamental cell biological processes of pregnancy. Autophagy is a dynamic process that is not only involved in the process of cell death, but also plays a role in cellular survival capacity to maintain cellular integrity.[11, 30, 31] Failure of autophagy in maintaining the integrity of a cell will result in acceleration of the processes of uncontrolled cell death, as found in variations of pathological pregnancies. The results of the study reveal that normal pregnancy as well as late-onset preeclampsia present better cell survival strategies than IUGR and especially early-onset preeclampsia.

## Conclusions

There were differences in the expression levels of the autophagy proteins LC3B and Beclin-1 as well as in the ratio of LC3B/Beclin-1 between the four different groups. Early-onset preeclampsia and IUGR had the highest expression, while normal pregnancy and late-onset pregnancy had highest LC3B/Beclin-1 ratios. There was a correlation between autophagy defects and free LDH as cell death marker. Early-onset preeclampsia had the highest negative correlation with LDH. Hence, autophagy plays a critical role as part of the cell death and cellular survival spectrum of villous trophoblast in late gestation bridging them to alterations in trophoblast deveopment early in pregnancy.

## Supporting information

S1 File Master data of research subject. (XLSX)

S2 File Compiled data of placenta quantification. (XLSX)

S3 File Raw data (1) LC3 of placenta quantification. (XLS)

S4 File Raw data (2) Beclin-1 of placenta quantification. (XLS)
